# Decoding single-cell molecular mechanisms in astrocyte-to-iN reprogramming via *Ngn2*- and *Pax6*-mediated direct lineage switching

**DOI:** 10.1186/s40001-024-01989-z

**Published:** 2024-07-27

**Authors:** Rongxing Qin, Yingdan Zhang, Yue Yang, Jiafeng Chen, Lijuan Huang, Wei Xu, Qingchun Qin, Xiaojun Liang, Xinyu Lai, Xiaoying Huang, Minshan Xie, Li Chen

**Affiliations:** 1 Department of Neurology, The First Affiliated Hospital of Guangxi Medical University, Nanning, 530021 Guangxi China; 2 National Center for International Biotargeting Theranostics, Guangxi Key Laboratory of Biotargeting Theranostics, Collaborative Innovation Center for Targeting Tumor Theranostics, Guangxi Medical University, Nanning, 530021 China

## Abstract

**Background:**

The limited regenerative capacity of damaged neurons in adult mammals severely restricts neural repair. Although stem cell transplantation is promising, its clinical application remains challenging. Direct reprogramming, which utilizes cell plasticity to regenerate neurons, is an emerging alternative approach.

**Methods:**

We utilized primary postnatal cortical astrocytes for reprogramming induced neurons (iNs) through the viral-mediated overexpression of the transcription factors *Ngn2* and *Pax6* (NP). Fluorescence-activated cell sorting (FACS) was used to enrich successfully transfected cells, followed by single-cell RNA sequencing (scRNA-seq) using the 10 × Genomics platform for comprehensive transcriptomic analysis.

**Results:**

The scRNA-seq revealed that NP overexpression led to the differentiation of astrocytes into iNs, with percentages of 36% and 39.3% on days 4 and 7 posttransduction, respectively. CytoTRACE predicted the developmental sequence, identifying astrocytes as the reprogramming starting point. Trajectory analysis depicted the dynamic changes in gene expression during the astrocyte-to-iN transition.

**Conclusions:**

This study elucidates the molecular dynamics underlying astrocyte reprogramming into iNs, revealing key genes and pathways involved in this process. Our research contributes novel insights into the molecular mechanisms of NP-mediated reprogramming, suggesting avenues for optimizing the efficiency of the reprogramming process.

## Background

In adult mammals, the inability of damaged neurons to regenerate severely limits neural repair. Although stem cell transplantation holds promise, clinical translation remains challenging [1]. Direct lineage reprogramming, which exploits cellular plasticity for neural regeneration, has emerged as an innovative alternative [2–6]. This method converts resident astrocytes into neurons, capitalizing on their similarity to progenitor cells or neural stem cells [7]. Transcription factors such as *Ngn2*, *Ascl1*, and *Dlx2* can directly differentiate astrocytes from the mouse cerebral cortex into neurons [8, 9].

Previous studies have indicated that reprogramming, driven by factors such as *Ascl1*, *Brn2*, and *Myt1l*, entails epigenetic changes influencing cell fate. For example, *Ascl1* initiates rapid chromatin remodeling and nucleosome repositioning, enabling fibroblasts to transform into induced neurons (iNs) within 12 h [10, 11]. *Myt1l* also plays a crucial role by inhibiting somatic differentiation and promoting neuronal function [12]. Despite these advances, the transcriptional mechanisms underlying direct neuronal fate conversion are not fully understood.

In this study, we utilized single-cell RNA sequencing (scRNA-seq) to investigate the molecular transcriptional mechanisms underlying the transformation of cultured astrocytes into iNs. This research may reveal the underlying mechanisms involved and provide a more in-depth understanding of astrocyte reprogramming.

## Materials and methods

### Primary culture of postnatal cortical astrocytes

We cultured postnatal astrocytes using a previously described method [13]. After removing the meninges, we dissected and mechanically dissociated cortical tissue from the cerebral cortex of P5‒P7 *C57BL/6 J* mice. The cells were then centrifuged at 1000 rpm for 2 min, resuspended, and cultured in medium containing DMEM/F12, 10% fetal bovine serum, 5% horse serum, and penicillin/streptomycin. To remove any contaminating oligodendrocyte precursor cells, the culture flasks were vigorously shaken multiple times. The cells were passaged once they reached 90% confluence. The experimental protocol received ethical approval from the Ethics Committee of Guangxi Medical University.

### Viral transduction of *Ngn2* and *Pax6*

First, we determined the optimal multiplicity of infection (MOI) value as 20 through gradient infection experiments. Subsequently, we coated a 24-well plate with a 0.1 mg/ml poly-D-lysine (PDL) solution and seeded astrocytes at a density of 1 × 10^^4^ cells per well. On the day of infection, the cells were divided into a control group and an overexpression group. The control group was infected with a mixture of Ubi-MCS-SV40-EGFP and Ubi-MCS-SV40-mCherry viruses, while the overexpression group was infected with a mixture of Ubi-MCS-SV40-Ngn2-EGFP and Ubi-MCS-SV40-Pax6-mCherry viruses. Sixteen hour postinfection, we replaced the medium with differentiation medium containing 20 ng/mL BDNF and subsequently replaced the medium every four days to maintain cell growth and viral expression. Viruses were directly purchased from Shanghai Genechem Co., Ltd.

### Fluorescence-activated cell sorting (FACS)

FACS was utilized to selectively enrich and collect cells that had been successfully transfected. The cells were trypsinized using 0.25% trypsin, followed by centrifugation. After centrifugation, the cells were resuspended in PBS (pH 7.4) at a concentration of 1 × 10^^6^ cells/mL, supplemented with 0.5% fetal bovine serum, and then placed on ice for further processing. We specifically targeted eGFP + or mCherry + cells, as indicated by the virus labeling, ensuring the accurate selection of the transfected cells, characterized by fluorescence. Using a FACS instrument, we enriched and collected the green (eGFP +) or red (mCherry +) fluorescent cells based on their fluorescence intensity, thereby ensuring high purity of the selected cell populations for subsequent downstream applications.

### Single-cell suspension preparation

After sampling, tissue fragments were promptly transferred to a centrifuge tube and incubated with trypsin at 37 °C for 10 min. The tube was then centrifuged at 1000 rpm for 2 min. The cells were resuspended in resuspension buffer consisting of 2.5 ml fetal bovine serum (FBS) and 47.5 ml phosphate-buffered saline (PBS). The cell suspension was passed through a flow cytometry cell strainer into a flow cytometry tube for cell sorting.

### 10 × Genomics library construction and sequencing

Cellular suspensions were processed using a 10X Genomics GemCode single-cell instrument to generate gel bead-in-EMlusions (GEMs). Libraries were constructed and sequenced from cDNA using Chromium Next GEM Single Cell 3’ Reagent Kits v3.1. Within each GEM, primers carrying an Illumina® R1 sequence, a 16 nt 10 × Barcode, a 10 nt UMI, and a poly-dT primer sequence were released and mixed with the cell lysate and Master Mix to reverse transcribe barcoded, full-length cDNAs from polyadenylated mRNA. The post-GEM reaction mixture was purified by silane magnetic beads, which removed residual reagents and primers. The full-length, barcoded cDNAs were subjected to PCR amplification, yielding sufficient material for library construction.

The R1 primer sequence was added to the molecules during GEM incubation. P5, P7, a sample index, and the R2 primer sequence were incorporated during library construction through processes such as end repair, A-tailing, adaptor ligation, and PCR. The final libraries contained the P5 and P7 primers utilized for Illumina bridge amplification. The P5 and P7 primers were contained in the resulting libraries and were compatible with Illumina sequencing. The standard Illumina paired-end constructs, which begin and end with P5 and P7, comprise a single cell 3’ library.

In Read 1, the single cell 3′ 16 bp 10 × barcode and 10 bp UMI were encoded, while Read 2 was utilized to sequence the cDNA fragment. The sample index sequences were integrated as the i7 index read. Both Read 1 and Read 2, serving as standard Illumina^®^ sequencing primer sites, were used for paired-end sequencing.

### scRNA-seq analysis

The Seurat package [14] was utilized to convert 10 × scRNA-seq data into a Seurat object, and the NormalizeData function was employed to normalize the expression matrix. A set of highly variable genes, totaling two thousand, was identified by using the FindVariableFeatures function. To reduce the dimensionality of the data set, principal component analysis (PCA) was performed using the RunPCA method. Further dimensionality reduction and cluster identification were achieved through the uniform manifold approximation and projection (UMAP) algorithm [15]. Significant marker genes for different clusters were identified using the FindAllMarkers function. For the annotation of cell types, marker genes from the literature were used in conjunction with the PanglaoDB database for annotation [16].

### Trajectory analysis

We revealed the differentiation trajectories and subtype evolution in the process of cell reprogramming by combining four methods. Gene expression changes were analyzed by the Monocle 2 algorithm using DDRTree technology and pseudotime series [17]. The VECTOR algorithm segments the UMAP into pixels, calculated PC value QP scores, identified developmental origins, and computed pseudotime scores [18]. Monocle 3 was used to sort cells based on pseudotime [19]. The dynamic process of cell state transition was demonstrated through pseudotemporal heatmaps. CytoTRACE scores of astrocytes and iNs were computed using the R package CytoTRACE [20], with a range from 0 to 1. Higher scores indicated less differentiation.

### High-dimensional weighted gene coexpression network analysis (hdWGCNA)

To explore the molecular functions of iNs, we utilized hdWGCNA to detect genes within coexpression modules that exhibited high levels of aggregation [21]. We applied the TestSoftPowers function to determine an optimal soft-power threshold (β) that facilitated the formation of a scale-free topological network. Subsequently, within each coexpression module, we pinpointed hub genes utilizing eigengene-based connectivity measures to establish their central roles within the network.

### Functional enrichment analysis

We utilized the ClusterProfiler R package to perform Gene Ontology (GO) analysis, and Kyoto Encyclopedia of Genes and Genomes (KEGG) pathway enrichment analysis.

### Statistical analysis

Statistical analyses were conducted using R (version 4.3.1). Gene expression comparisons between the two groups were assessed using the Wilcoxon test. Differences were considered significant when *p* < 0.05.

## Results

### Single-cell atlas

**Fig. 1:**
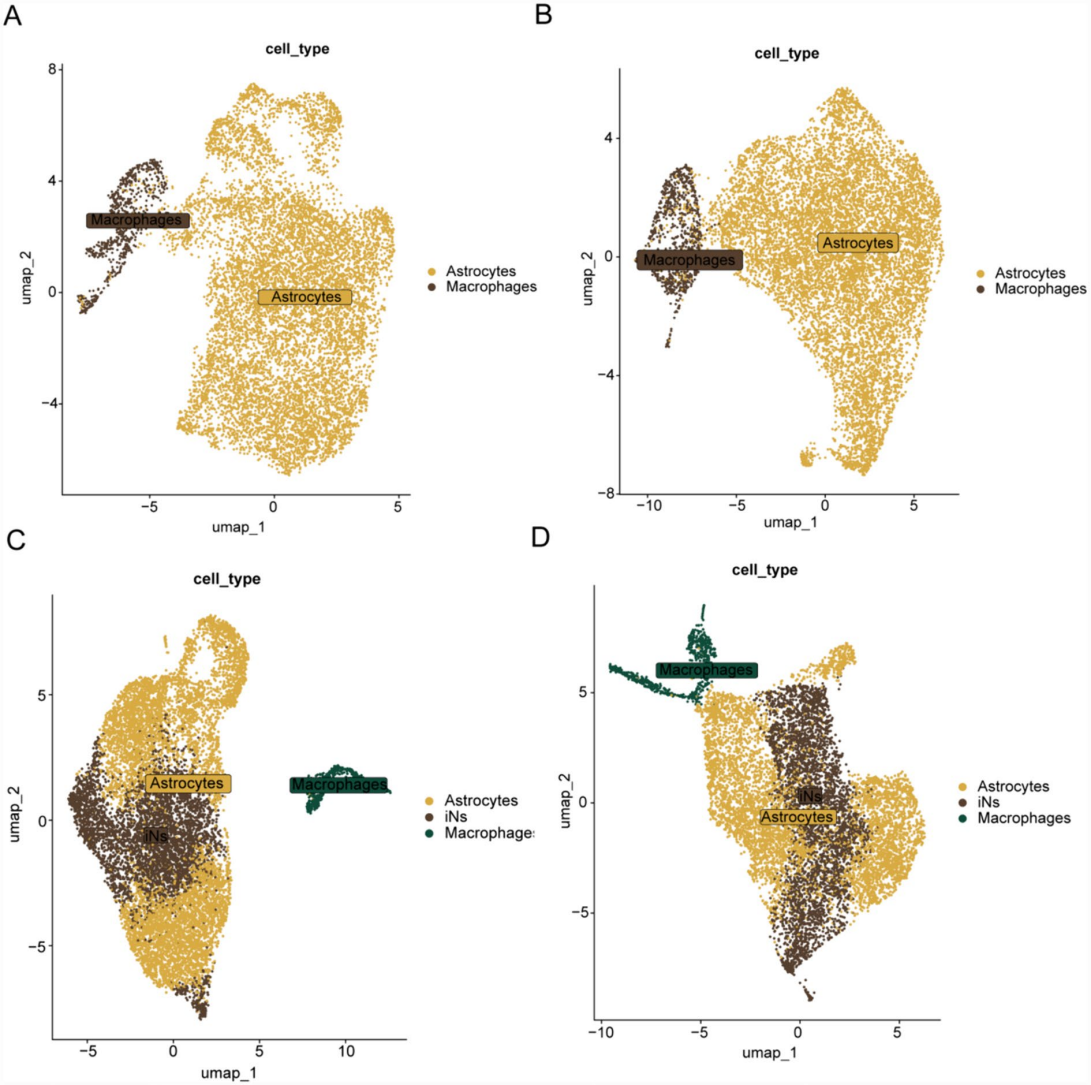
Classification of cell types among the four groups: **A** UMAP of D4-C, **B** UMAP of D7-C, **C** UMAP of D4-OE, and **D** UMAP of D7-OE. UMAP, uniform manifold approximation and projection

To explore the molecular mechanisms of astrocyte reprogramming, we performed single-cell sequencing analysis on cultured astrocytes. During the early stages of reprogramming, specifically on days 4 and 7 after lentivirus transduction, we isolated astrocytes from the cortex of neonatal mice using FACS and performed high-throughput scRNA-seq. The purpose of this experiment was to evaluate the proportion of iNs in the following groups: the control group on day 4 (D4-C), the control group on day 7 (D7-C), the *Ngn2* and *Pax6* (NP) overexpression group on day 4 (D4-OE), and the NP overexpression group on day 7 (D7-OE). The results showed the absence of iNs in the D4-C and D7-C groups. The incidence of iNs was 36% and 39.3% in the D4-OE and D7-OE groups, respectively (Fig. 1).

### CytoTRACE and expression of marker genes

**Fig. 2:**
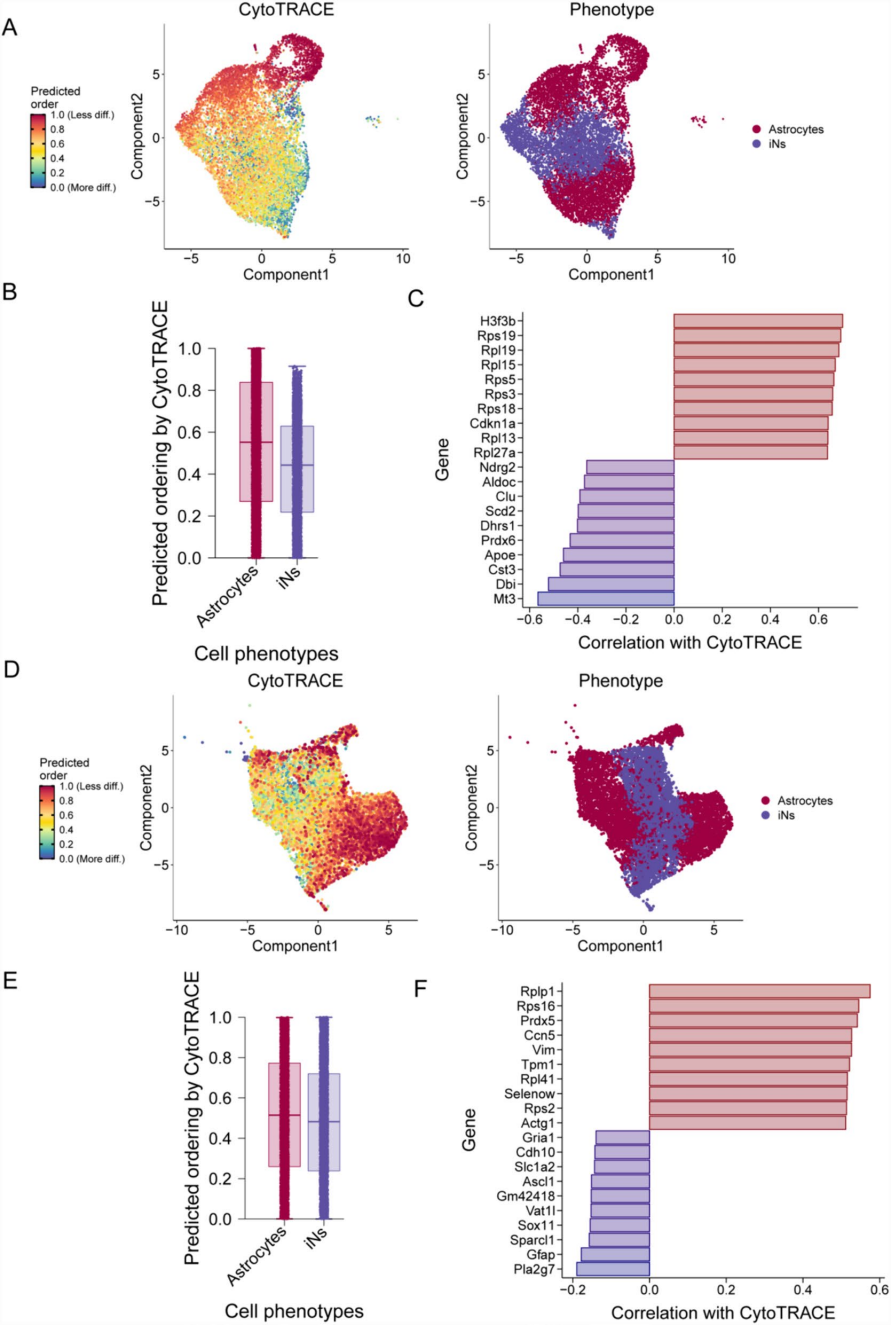
Stemness of astrocytes and iNs was assessed using CytoTRACE to predict the cell differentiation potential of both cell types. **A**, **B** tSNE plot and boxplot depicting the distribution of CytoTRACE scores among astrocytes and iNs in the D4-OE group, where dark green indicates lower scores (low stemness) and dark red indicates higher scores (high stemness). **C** Genes correlated with cell differentiation processes in the D4-OE group, as predicted by CytoTRACE. **D**, **E** tSNE plot and boxplot showing the distribution of CytoTRACE scores among astrocytes and iNs in the D7-OE group, with dark green indicating lower scores (low stemness) and dark red indicating higher scores (high stemness). **F** Genes correlated with cell differentiation processes in the D7-OE group as predicted by CytoTRACE. tSNE, t-distributed dtochastic neighbor embedding

In this study, we applied the CytoTRACE approach to predict developmental sequences and starting points. In the D4-OE group, the results showed that astrocytes had the highest CytoTRACE scores, indicating a low degree of differentiation and suggesting that they were at the starting point of the cell trajectory (Fig. 2A, B). Additionally, we discovered genes correlated with cell differentiation processes in the D4-OE group, such as *H3f3b* and *Cdkn1a* (Fig. 2C). Similarly, in the D7-OE group, astrocytes were at the starting point of the cell trajectory (Fig. 2D, E). Furthermore, we identified genes correlated with cell differentiation processes in the D7-OE group, such as *Prdx5* and *Gfap* (Fig. 2F).

**Fig. 3:**
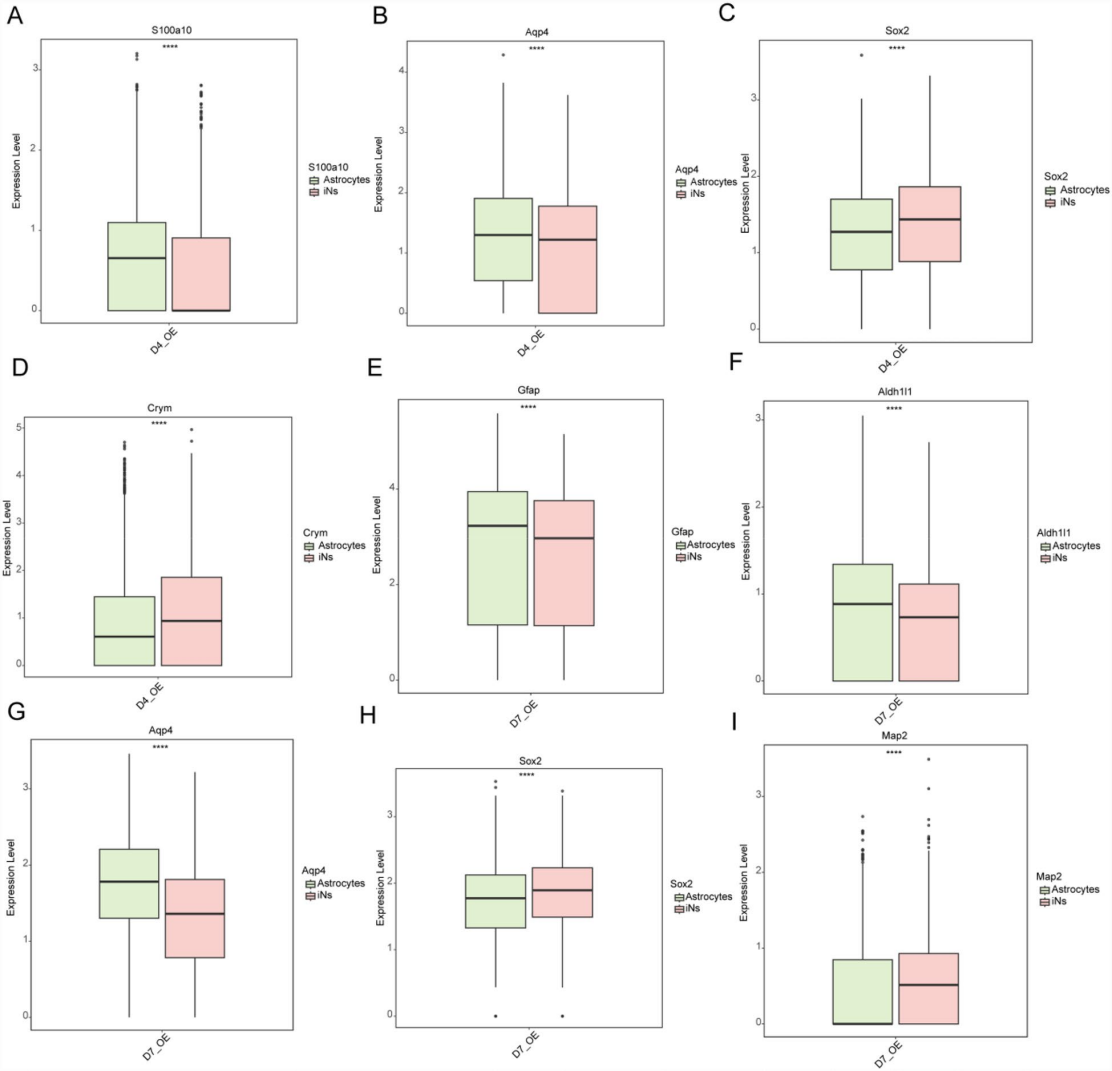
Differential expression of astrocytic and neuronal markers in iNs and astrocytes across the D4-OE and D7-OE groups. **A**, **B** Expression of the astrocytic markers *S100a10* and *Aqp4* in the D4-OE group. **C**–**D** Expression of the neuronal markers *Sox2* and *Crym* in the D4-OE group. **E**–**G** Expression of the astrocytic markers *Gfap*, *Aldh1l1*, and *Aqp4* in the D7-OE group. **H**, **I** Expression of neuronal *Sox2* and *Map2* in the D7-OE group. ****p < 0.0001

In our study, we examined the expression of astrocytic and neuronal markers in the NP group, focusing on the differences between astrocytes and iNs. Compared to astrocytes iNs in the D4-OE group, exhibited a reduced expression of the astrocytic markers *S100a10* and *Aqp4* (Fig. 3A, B). Concurrently, these iNs displayed elevated expression of the neuronal markers *Sox2* and *Crym* (Fig. 3C, D). Similarly, in the D7-OE group, compared with astrocytes, iNs exhibited decreased expression of the astrocytic markers *Gfap*, *Aldh1l1*, and *Aqp4* (Fig. 3E–G). In contrast, these iNs exhibited increased expression levels of the neuronal markers *Sox2* and *Map2* (Fig. 3H, I).

### Trajectory analysis of marker genes

**Fig. 4:**
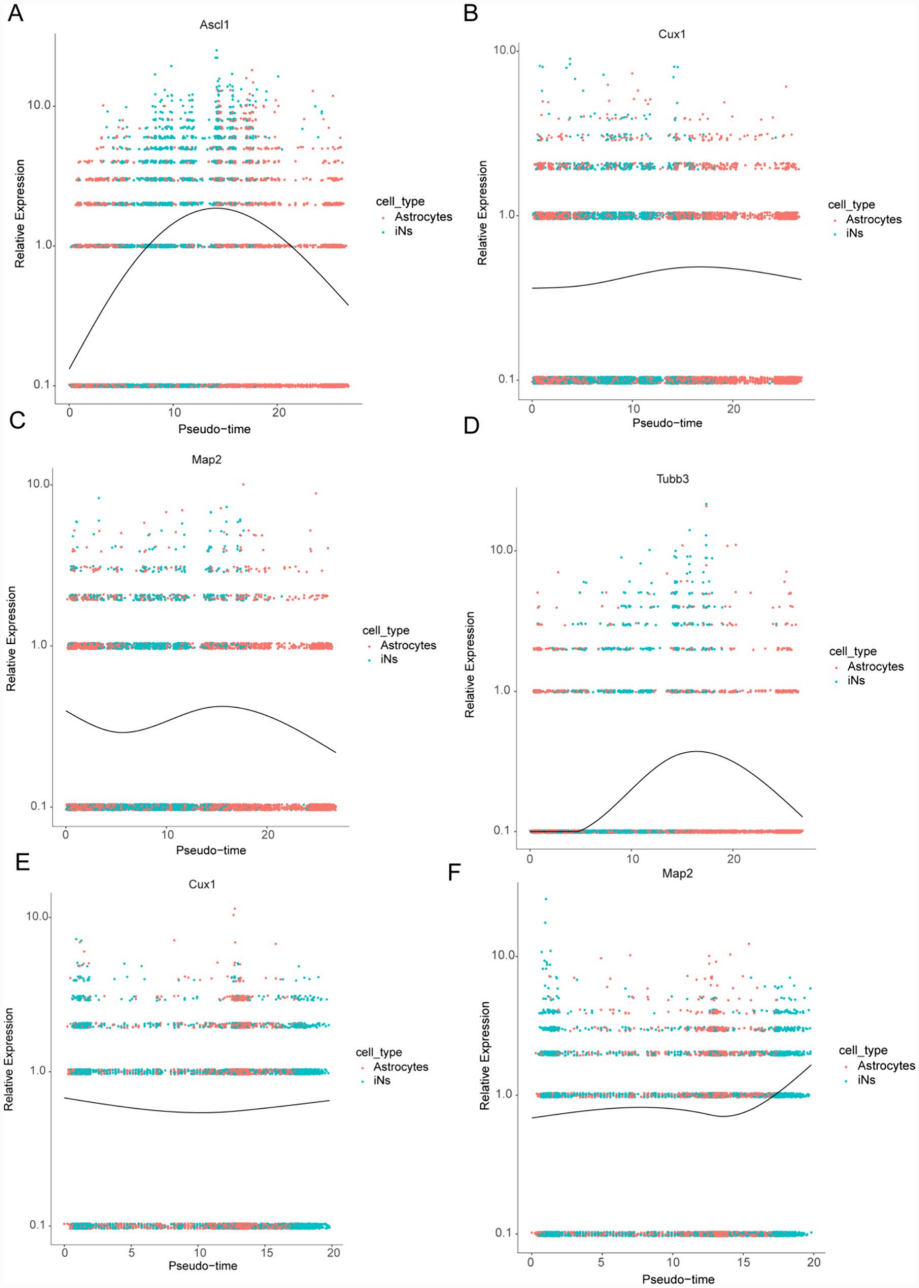
Relative expression profiles of marker genes in the differentiation pseudotime trajectory. **A**–**D** D4-OE. A, Expression analysis of *Ascl1*; **B** Expression analysis of *Cux1*, **C** Expression analysis of *Map2*; **D** Expression analysis of *Tubb3*. **E**, **F** D7-OE. **E** Expression analysis of *Cux1*; **F** Expression analysis of *Map2*

To deepen our understanding of astrocyte differentiation mechanisms, we performed a dynamic gene expression analysis utilizing Monocle 2. This analysis revealed temporal shifts in gene expression as astrocytes transitioned to iNs. Specifically, in the D4-OE group, during the process of differentiation, we observed the upregulation of *Ascl1*, *Cux1*, *Map2*, and *Tubb3* in the early stages (Fig. 4A–D). Similarly, in the D7-OE group, *Cux1* and *Map2* exhibited increased expression levels during the transformation process of astrocytes compared to those at the beginning of the transformation (Fig. 4E, F).

### Pseudotemporal heatmap and functional enrichment analysis

**Fig. 5:**
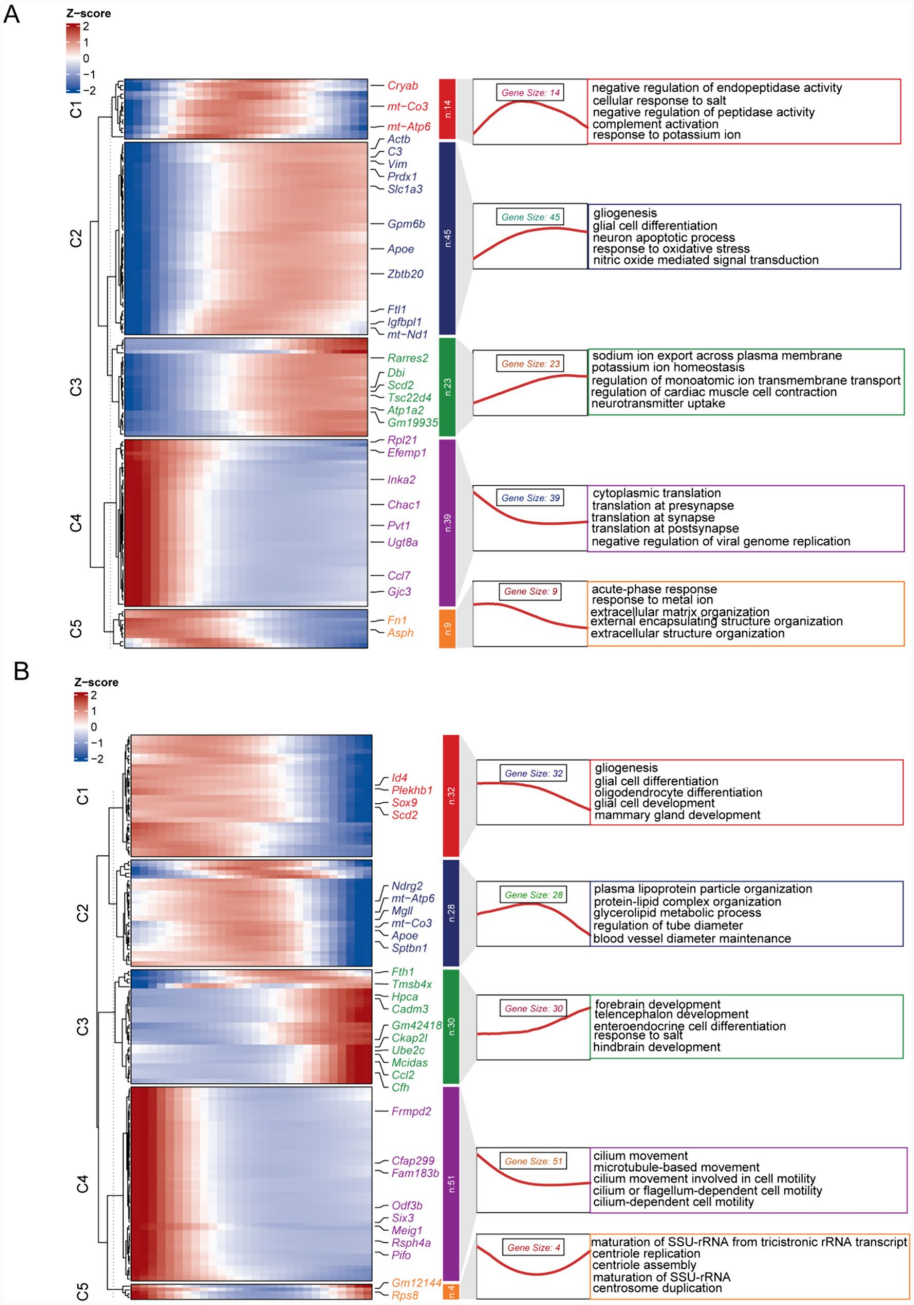
Heatmap illustrating gene expression patterns over pseudotime and functional enrichment analysis of genes throughout the astrocyte-to-iN conversion process. **A** D4-OE; **B** D7-OE

We utilized pseudotemporal heatmaps to analyze the temporal dynamics of gene expression throughout the astrocyte-to-iNs transition. We detected concurrent activation of genes such as *Prdx1* and *Slc1a3* in the D4-OE stage. Functional enrichment analysis indicated that these activated genes were predominantly involved in pathways such as glial cell differentiation, neuron apoptotic process, response to oxidative stress and neurotransmitter uptake (Fig. 5A).

Furthermore, in the D7-OE stage, we detected an upregulation of genes, including *Fth1*, *Tmsb4x*, *Hpca*, *Cadm3*, and *Gm42418*, as astrocytes converted into iNs. Functional enrichment analysis revealed enrichment of genes involved in forebrain development, telencephalon development, hindbrain development and centrosome duplication (Fig. 5B).

### High-dimensional weighted gene coexpression network analysis (hdWGCNA) and functional enrichment analysis

**Fig. 6:**
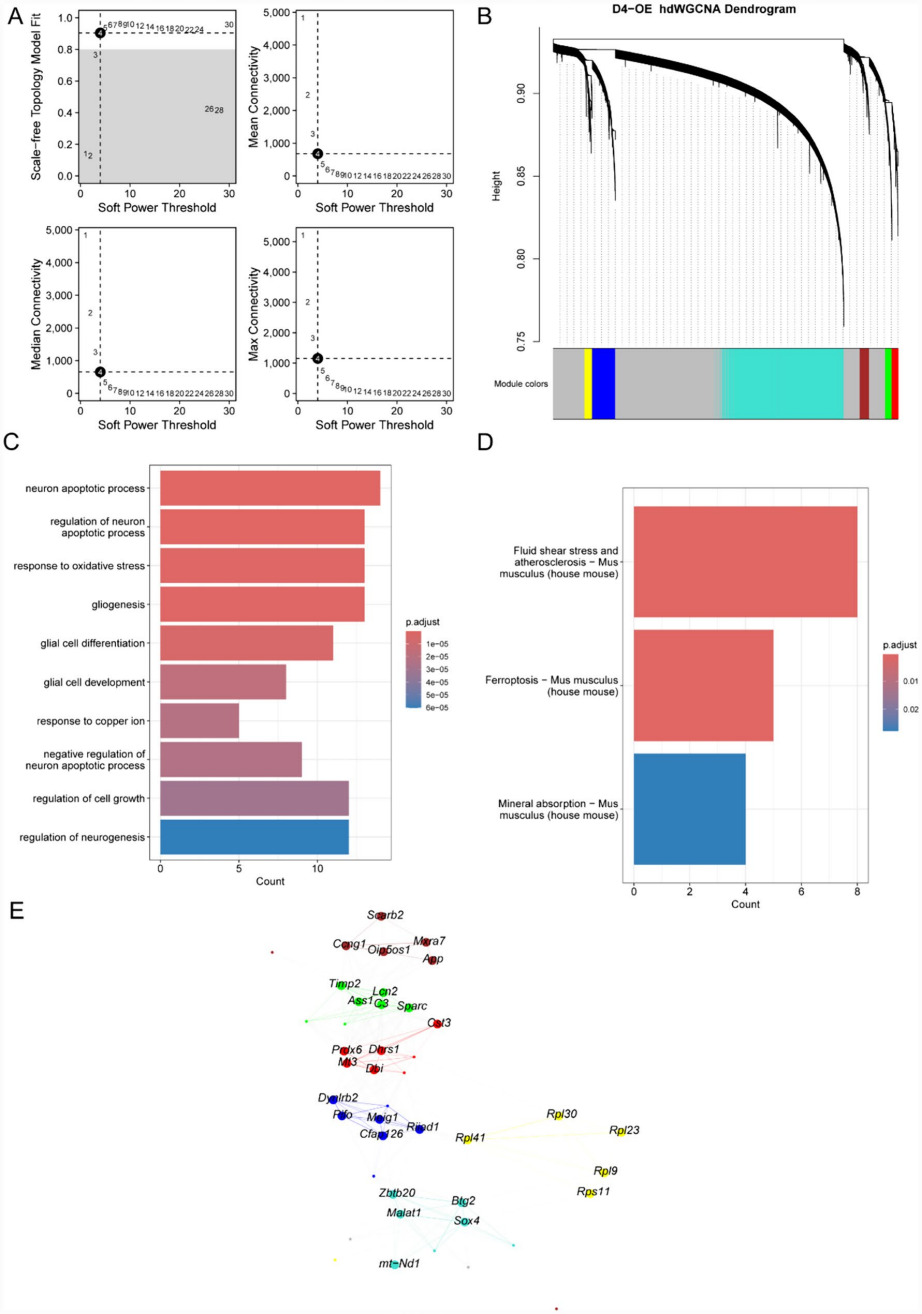
hdWGCNA revealed hub genes in the iNs module of the D4-OE group. **A** Fit indices of the scale-free topology model and the average connectivity were evaluated for different soft-thresholding powers. After careful analysis, the optimal soft threshold was determined to be 4. **B** iNs were subjected to high dimensional weighted gene coexpression analysis. **C** GO analysis of the hub genes associated with the iNs module. **D** KEGG analysis of the hub genes associated with the iNs module. **E** Visualization of hub genes associated with the iNs module

We constructed a weighted coexpression network to detect gene functional modules, as many genes exert their effects through close interactions and exhibit similar expression patterns. We used hdWGCNA to identify the major molecular features of iNs on D4-OE. With a soft threshold of 4, we constructed a scale-free network of iNs to obtain optimal connectivity and identified 7 gene modules (Fig. 6A, B). We performed GO enrichment analysis on the hub genes and found that their functions were mainly related to glial cell differentiation, regulation of cell growth, and regulation of neurogenesis (Fig. 6C). KEGG pathway enrichment analysis revealed significant enrichment in pathways associated with ferroptosis, fluid shear stress and atherosclerosis, and mineral absorption (Fig. 6D). We also identified the top five hub genes of iNs on D4-OE (Fig. 6E).

**Fig. 7:**
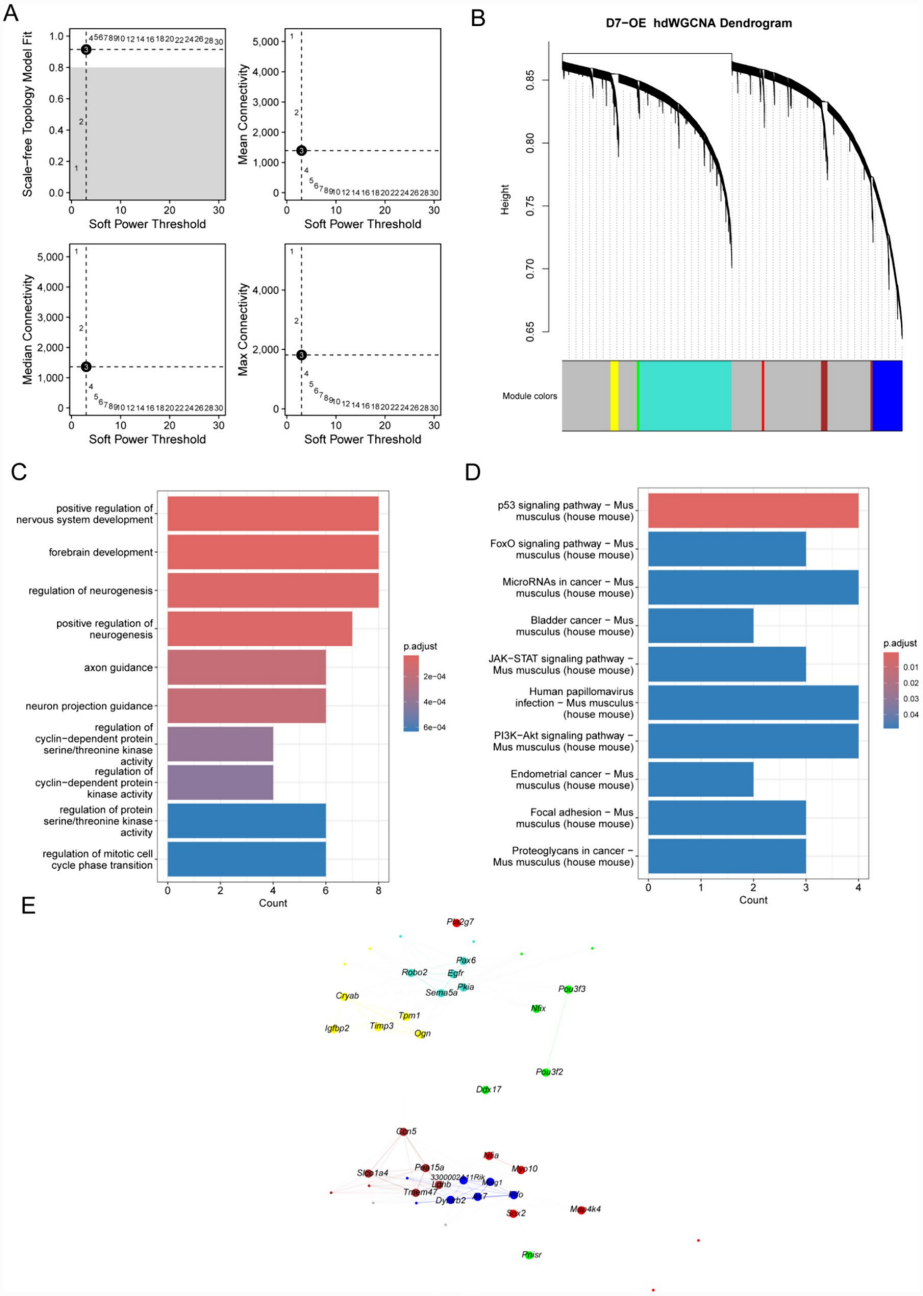
hdWGCNA revealed hub genes in the iNs module of the D7-OE group. **A** Fit indices of the scale-free topology model and the average connectivity were evaluated for different soft-thresholding powers. After careful analysis, the optimal soft threshold was determined to be 3. **B** iNs were subjected to high dimensional weighted gene coexpression analysis. **C** GO analysis of the hub genes associated with the iNs module. **D** KEGG analysis of the hub genes associated with the iNs module. **E** Visualization of hub genes associated with the iNs module

Furthermore, we identified the major molecular features of iNs on D7-OE. With a soft threshold of 3, we constructed a scale-free network of iNs and identified 7 gene modules (Fig. 7A, B). We performed GO enrichment analysis on the hub genes and found that their functions involved mainly positive regulation of nervous system development, regulation of neurogenesis, positive regulation of neurogenesis, axon guidance, and neuron projection guidance (Fig. 7C). KEGG pathway enrichment analysis revealed that the main enriched pathways were the JAK − STAT signaling pathway and the PI3K–Akt signaling pathway (Fig. 7D). We also identified the top five hub genes of iNs on D7-OE (Fig. 7E).

## Discussion

In this study, compared with those in control astrocytes, the proportions of iNs generated from NP-mediated astrocytes in the D4-OE and D7-OE groups were 36% and 39.3%, respectively. Dynamic gene expression during the conversion process was described, and it was found that the expression of certain neuronal markers (*Map2*, *Cux1*) gradually increased during the conversion. Through pseudotemporal heatmaps, we observed that during astrocyte-to-iN conversion, a set of genes were simultaneously activated in D4-OE cells, participated in glial cell differentiation and neurotransmitter uptake. On the other hand, in D7-OE, another set of genes was simultaneously activated, involving pathways such as forebrain development, telencephalon development, hindbrain development and centrosome duplication. By constructing a weighted coexpression network, we determined the major molecular features of iNs during D4-OE and D7-OE. On D4-OE, genes related to glial cell differentiation, regulation of cell growth, and regulation of neurogenesis were associated with iNs. Moreover, in D7-OE, genes related to the positive regulation of nervous system development, regulation of neurogenesis, positive regulation of neurogenesis, axon guidance, and neuron projection guidance were associated with iNs. These results revealed the molecular characteristics and regulatory mechanisms underlying the astrocyte-to-iN conversion.

*Nng2* is essential for the glutamatergic neurotransmitter phenotype in the embryonic neocortex and effectively converts astrocytes into glutamatergic neurons, underscoring its role in excitatory neurogenesis. Additionally, *Ngn2* induces both excitatory and inhibitory neurons in the spinal cord [22]. Through the CRISPRa system, the activation of *Ngn2* and *Isl1* can convert astrocytes into functional motor neurons [23]. Furthermore, when *Ngn2* combines with *Nurr1*, it can induce the formation of dopaminergic neurons [24]. As a key regulator of neurogenesis, *Pax6* serves as an intrinsic determinant of the neurogenic potential of glial cells [13]. *Pax6* transduction in proliferative glial cells induces the expression of early neuronal markers [25].

This study employed the CytoTRACE method, a novel approach to delineate the developmental trajectory and pinpoint the initiation points of astrocytes as they undergo reprogramming into iNs. Our findings in the D4-OE and D7-OE groups revealed that astrocytes with elevated CytoTRACE scores are less differentiated, supporting the premise that these cells are poised at the onset of their cellular developmental pathway. Genes associated with cell differentiation processes, such as *H3f3b*, *Cdkn1a*, and *Prdx5*, were identified. *H3f3b*, the gene encoding histone H3.3, is crucial for establishing the identity of mitotic neurons after key developmental stages [26]. *CDKN1A*/p21, a cyclin-dependent kinase inhibitor, plays a pivotal role in the suppression of ferroptosis through its induction by p53 [27]. This is particularly relevant in the context of neuronal reprogramming, where the generation of reactive oxygen species (ROS) has been reported to impede the reprogramming process [28].

Additionally, *PRDX5*, an antioxidant protein, is associated with aberrant ROS [29]. Given that oxidative stress-induced ferroptosis can impose limitations on neuronal reprogramming [30], the potential of *PRDX5* as a key mediator in safeguarding cells against ROS, particularly those originating from mitochondrial sources, is highlighted [31]. These insights collectively enhance our understanding of the molecular dynamics during the reprogramming of astrocytes into iNs and offer avenues for developing targeted strategies to optimize this process.

The reprogramming of astrocytes into iNs is a complex and intricate process that involves a delicate interplay of transcription factors and cellular markers, which are pivotal in determining cell fate. Following the overexpression of NP in astrocytes, we observed a rapid downregulation of astrocytic genes in both the D4-OE and D7-OE groups. The rapid transcriptomic changes observed after overexpressing neural transcription factors are consistent with previous reports on *NeuroD1*, *Ascl1*, or *Ngn2* [10, 32–34]. The observed downregulation of astrocytic markers such as *S100a10*, *Aqp4*, and *Gfap*, coupled with the upregulation of neuronal markers such as *Sox2*, *Map2*, and *Crym*, underscores the phenotypic metamorphosis that astrocytes undergo as they are reprogrammed into iNs. As revealed by our Monocle 2 analysis, the temporal dynamics of gene expression provided deeper insights into the molecular timeline of the reprogramming process from astrocytes to iNs. There was early upregulation of *Ascl1*, *Cux1*, *Map2*, and *Tubb3* in the D4-OE group, with increased expression of *Map2* and *Cux1* in the D7-OE group during the transformation process. Previous studies have shown that the direct reprogramming of fibroblasts into neurons involves two stages: an initiation stage in which *Ascl1* induces neuronal fate, and a maturation stage in which reprogrammed fibroblasts permanently acquire a neuronal identity [12]. This may also hold true for astrocytes, as *Ascl1* expression increases and then decreases during the process of astrocyte-to-iN differentiation. In the initial stage, *Ascl1* expression increases, and *Ascl1* functions as a core driver of reprogramming [10], with its overexpression capable of inducing rapid neuronal differentiation [35]. * Map2*, which is predominantly expressed in neurons, is integral to axonal and dendritic growth, synaptic plasticity, and overall neuronal development [36]. *Cux1* promotes the integration of layer II–III neurons in the cortical network in a highly specific manner [37]. The orchestrated modulation of astrocytic and neuronal markers during the reprogramming of astrocytes into iNs highlights the complexity of cell fate determination. Understanding the molecular choreography that drives this process is vital for harnessing the therapeutic potential of cellular reprogramming in neurological applications.

The pseudotemporal heatmaps allowed us to dissect the temporal dynamics of this transition, revealing significant insights into the genes and pathways involved. In the D4-OE stage, the activation of genes such as *Prdx1* and *Slc1a3* is noteworthy. Antioxidant proteins play a crucial role in maintaining body homeostasis. *PRDX1* possesses an antioxidant function and is capable of clearing ROS within the body [38]. It interacts with various kinases and enzymes and can prevent cell apoptosis caused by oxidative stress [39]. Its upregulation during the astrocyte-to-iN transition suggests a potential protective mechanism against oxidative stress, which is known to be a critical factor in neurogenesis [40]. Glutamate levels are regulated by *SLC1A3*, which reduces excitotoxicity and helps to prevent damage to the nervous system caused by excitotoxicity [41]. The functional enrichment analysis revealed that these activated genes are predominantly involved in pathways associated with glial cell differentiation, neuron apoptotic process, response to oxidative stress and neurotransmitter uptake. Moreover, direct neuronal reprogramming to obtain neurons is a process that can induce oxidative stress [42], which necessitates an appropriate response to oxidative stress to maintain cellular homeostasis and ensure the successful differentiation of reprogrammed cells.

In the subsequent D7-OE stage, genes such as *Fth1*, *Tmsb4x*, *Hpca*, *Cadm3*, and *Gm42418* were upregulated. *TMSB4X* plays a crucial role in the transdifferentiation of human fibroblasts into myogenic cells, enhancing the efficiency of the process [43]. The expression level of *HPCA* increases during neuronal differentiation, and overexpression of *HPCA* enhances neuronal differentiation [44]. *CADM3* mediates direct contact and interaction between axons and glial cells [47].

The hdWGCNA was used to identify gene modules and hub genes that play a central role in the reprogramming process. GO enrichment analysis of the hub genes in the D7-OE group revealed functions that are essential for the development and maturation of the nervous system, including positive regulation of nervous system development, regulation of neurogenesis, axon guidance, and neuron projection guidance. Neurons undergo processes such as neuronal migration, axonal elongation, axon pruning, dendritic morphogenesis, synaptic maturation, and plasticity to form neural circuits [45]. Moreover, KEGG pathway enrichment analysis revealed pathways that are implicated in neurodegenerative diseases, such as ferroptosis, and other pathways related to the JAK–STAT and PI3K–Akt signaling pathways. The JAK–STAT3 pathway is not only recognized as the core signaling pathway for maintaining pluripotency but also has recently been shown to be essential for the complete reprogramming of mouse somatic cells [46]. The dedifferentiation of astrocytes into an undifferentiated state is induced through the activation of the PI3K/Akt/p21 signaling pathway, thereby promoting their transdifferentiation into neurons [48].

There are limitations to this study. Our research is based on in vitro experiments, and the results need to be validated in vivo to ensure their reliability and reproducibility. The specific mechanisms and processes of transformation still require further investigation. There may be unknown factors and obstacles during the cellular conversion process, which could affect the efficiency of conversion and the stability of cell characteristics. Our study revealed the potential of the NP to reprogram astrocytes into iNs, explored the molecular features and regulatory mechanisms of astrocyte-to-iN conversion.

## Conclusion

This study elucidated the molecular dynamics of astrocyte reprogramming into iNs by identifying key genes and pathways involved in this process. The CytoTRACE method revealed less differentiated astrocytes as the starting point for reprogramming. Overexpression of *Ngn2* and *Pax6* led to rapid downregulation of astrocytic genes and upregulation of neuronal markers, indicative of phenotypic transformation. This study also highlighted the role of antioxidants in countering oxidative stress during reprogramming, which is essential for maintaining cellular homeostasis and successful neuronal differentiation. Despite in vitro limitations, the findings offer insights into the regulatory mechanisms of astrocyte-to-iN conversion, potentially aiding in developing therapeutic strategies for neurodegenerative diseases.

## Data Availability

All the data generated or analyzed during this study are included in the methods section of this article. Other data that support the findings of this study are available from the corresponding author upon reasonable request.
